# The Role of microRNA-155 as a Biomarker in Diffuse Large B-Cell Lymphoma

**DOI:** 10.3390/biomedicines12122658

**Published:** 2024-11-21

**Authors:** Epameinondas Koumpis, Vasileios Georgoulis, Konstantina Papathanasiou, Alexandra Papoudou-Bai, Panagiotis Kanavaros, Evangelos Kolettas, Eleftheria Hatzimichael

**Affiliations:** 1 Department of Hematology, Faculty of Medicine, School of Health Sciences, University of Ioannina, 45500 Ioannina, Greece; an.koumpis@uoi.gr (E.K.); vasileios.georgoulis@gmail.com (V.G.); konppth@gmail.com (K.P.); 2 Department of Pathology, Faculty of Medicine, School of Health Sciences, University of Ioannina, 45500 Ioannina, Greece; apapoudoubai@gmail.com; 3 Department of Anatomy-Histology-Embryology, Faculty of Medicine, School of Health Sciences, University of Ioannina, 45110 Ioannina, Greece; pkanavar@uoi.gr; 4 Laboratory of Biology, Faculty of Medicine, School of Health Sciences, Institute of Biosciences, University Centre for Research and Innovation, University of Ioannina, 45110 Ioannina, Greece; ekoletas@uoi.gr; 5 Biomedical Research Institute, Foundation for Research and Technology, 45110 Ioannina, Greece; 6 Computational Medicine Center, Sidney Kimmel Medical College, Thomas Jefferson University, Philadelphia, PA 19107, USA

**Keywords:** lymphoma, DLBCL, miRNAs, miR-155, biomarkers, lymphomagenesis, diagnostic, prognostic, predictive

## Abstract

Diffuse Large B-cell Lymphoma (DLBCL) is the most common aggressive non-Hodgkin lymphoma (NHL). Despite the use of newer agents, such as polatuzumab vedotin, more than one-third of patients have ultimately relapsed or experienced refractory disease. MiRNAs are single-stranded, ~22-nucleotide-long RNAs that interact with their target RNA. They are significant regulators of post-transcriptional gene expression. One significant miRNA, miR-155, is involved in the pathophysiology of DLBCL and it is a critical modulator of hematopoiesis, inflammation, and immune responses. Targets of miR-155, such as histone deacetylase 4 (HDAC4), suppressor of cytokine signaling-1 (SOCS1) and immune cells, play a crucial role in DLBCL pathogenesis, since miR-155 regulates key pathways, transcription factors and cytokine expression and shapes the tumor microenvironment in DLBCL. In this review, we examine the role of miR-155 in DLBCL and its potential as a future diagnostic, prognostic, or predictive biomarker.

## 1. Introduction

Diffuse large B-cell lymphoma (DLBCL) is an aggressive non-Hodgkin lymphoma (NHL) characterized by significant diversity in its molecular and pathological subtypes as well as in clinical manifestation, leading to different treatment responses and outcomes [1,2]. It is the most common NHL, with an increasing incidence [1,2]. Rituximab, an anti-CD20 monoclonal antibody, is a key treatment for DLBCL, since it specifically targets the CD20 protein found on the surface of B-cells. When rituximab was combined with CHOP (cyclophosphamide, doxorubicin, vincristine, and prednisolone) as a backbone, it led to a significant increase in 5-year overall survival (OS) (58% vs. 45%); thus, R-CHOP remained the standard first-line treatment for these patients until recently [3]. R-CHOP may cure 50–70% of patients [4]. However, 30% will ultimately relapse after having achieved complete remission (CR), while 20% will be proven to have primary refractory disease [5,6]. DLBCL can result from the transformation of a pre-existing indolent lymphoma, a slow-growing non-Hodgkin low-grade lymphoma, such as follicular lymphoma (FL). However, DLBCL usually arises without a history of lymphoma (“de novo”) [7].

Regarding the molecular background of the disease, gene expression profiling has identified three molecular subtypes of DLBCL: the germinal center B-cell–like (GCB) subtype, the activated B-cell–like (ABC) subtype, and the type 3 subtype or unclassified cases [8]. Non-GCB cases includes both ABC and unclassified cases. These molecular subtypes are believed to arise from different stages of lymphoid cell differentiation (cell of origin), while new taxonomies have further refined the classification based on detailed analyses of molecular and cytogenetic aberrations [2,9]. Specifically, a molecular classification of DLBCL that includes the following subtypes has been proposed: (a) the MCD (*MYD88L265P* and *CD79B* co-mutated) subtype, (b) the BN2 (*BCL6* fusions or *NOTCH2* mutated) subtype, (c) the N1 (*NOTCH1* mutated) genetic subtype, and (d) the *EZH2* mutated or BCL2 translocated (EZB) subtype; additionally, there is a large proportion of patients that remains unassigned [10]. Based on genomic analysis of 304 cases of DLBCL, a new molecular classification was also proposed that includes five clusters (C): C1, associated with *NOTCH2* mutations and favorable outcomes; C2, associated with aneuploidy, *TP53* biallelic inactivation, and poor outcomes, C3, which harbors *BCL2* mutations and translocations, as well as mutations in epigenetic modifiers, and it is associated with unfavorable outcomes; C4, with abnormalities affecting signaling pathways, such as Jak/STAT, and which is associated with favorable outcomes; and C5, which includes cases with 18q gains and *MYD88* and *CD79B* mutations and is associated with poor outcomes. In this study, they also assessed the prognostic value of various genetic features and among others, the gain of 13q31.3/miR-17-92 was independently predictive of inferior progression free survival (PFS), suggesting that alterations in microRNA (miRNA) expression are also important in DLBCL [11]. These new taxonomies that classify DLBCL into more specific subtypes based on genetic, molecular, and immunophenotypic characteristics aim to both better capture of DLBCL’s heterogeneity and to make DLBCL treatment more individualized and effective. However, the new taxonomies are derived from molecular techniques that may not yet be accessible in routine clinical practice or need further validation, and these may be the reasons why they have not yet been incorporated into the new classifications of hematolymphoid tumors published by either the World Health Organization (WHO) or by the International Consensus Classification (ICC) [12,13].

In relation to routine diagnostic histopathology, immunohistochemical algorithms such as the Hans, the Choi, and the Tally algorithms, using different combinations of antibodies to germinal center or ABC B-cell-related proteins (e.g., Bcl-6, CD10, MUM1, LMO2 and GCET1 antibodies), have been developed for the classification of DLBCL on the basis of the cell of origin being GCB or non-GCB [14,15,16].

In order to categorize patients with DLBCL into distinct risk groups and more accurately evaluate their prognosis, numerous scoring systems have been developed. The most commonly used is the International Prognostic Index (IPI), although it was devised before the advent of rituximab. This scoring system incorporates age, serum lactate dehydrogenase (LDH) levels, Ann Arbor disease stage, the patient’s European Cooperative Oncology Group (ECOG) performance status, and the extent of extranodal disease to stratify patients in four risk categories (low, low-intermediate, high-intermediate, or high) with decreasing estimated OS ranging from 91% 3-year OS for low-risk patients to 59% for high-risk ones [17]. The revised IPI (R-IPI) after the incorporation of rituximab in treatment regimens used the same risk factors but grouped patients in three categories, with the prognosis being termed as very good (no risk factor), good (1–2 risk factors), or poor (3–4 risk factors) [18]. The NCCN-IPI prognostic scoring system was developed for newly diagnosed patients with DLBCL aimed at being treated with R-CHOP [19]. This was derived from the National Comprehensive Cancer Network database and was generally based on the same adverse risk variables as the previous scores, but with further risk stratification, again allocating patients to four risk categories (low, low-intermediate, high-intermediate, high-risk). The NCCN-IPI seems to have the highest predicting capacity among the three scores, including PFS and OS; however, all three indices were still not able to identify a risk group with very poor prognosis, indicating an unmet need in the field of DLBCL risk stratification [20,21,22]. Recently, a novel prognostic index for patients with DLBCL was developed, the International Metabolic Prognostic Index, incorporating age, disease stage, and the baseline metabolic tumor volume (MTV) based on positron emission tomography (PET) imaging [23].

In order to enhance the prognosis assessment of patients with DLBCL, numerous research groups have investigated novel biomarkers. Circulating tumor DNA (ctDNA) has attracted substantial research interest among these biomarkers, particularly following initial investigations in patients with non-hematologic solid malignancies. ctDNA is fragmented DNA, with a length varying between 70 base pairs and 21 kilobases, which is released by neoplastic cells in the bloodstream via apoptosis, necrosis, or active release [24]. Several studies have demonstrated the potential role of ctDNA as a non-invasive biomarker for prognosis, monitoring of treatment response, and early detection of relapse in patients with DLBCL [25]. Regarding its contribution to determining patients’ prognosis, both pre-treatment and interim ctDNA levels have been shown to correlate with survival rates or the risk of disease progression either in previously untreated patients or in the salvage treatment setting [26,27,28]. A systematic review addressing the utility of ctDNA as a form of “liquid biopsy” in DLBCL concluded that IGH gene rearrangements and somatic mutations detected in ctDNA seem to be the most useful biomarkers to date in assessing treatment response, while the prognostic value of ctDNA concentration and methylation status is not clear and needs further investigation [29]. Interestingly, recent clinical trials of novel treatment agents, such as polatuzumab vedotin or odronextamab, have utilized ctDNA as a biomarker of molecular response. It has been observed that patients who test negative for ctDNA during therapy or at the end of treatment tend to have a longer survival [30,31]. ctDNA might, hence, assist in therapeutic decisions regarding the escalation or de-escalation of treatment when used at certain time points [22]. Recently, interest has increased in regard to various circulating biomarkers, including circulating tumor cells and different types of circulating-free RNA (cfRNA), as well as RNA encapsulated in extracellular vesicles (EVs) or platelets. These include coding RNAs, like messenger RNA (mRNA), and non-coding RNAs, such as miRNA and circular RNA (circRNA) [32].

MiRNAs belong to a large family of naturally occurring, endogenous, single-stranded ~22-nucleotide-long RNAs that interact with their target RNA in a sequence-dependent manner and therefore are significant regulators of post-transcriptional gene expression [33,34]. In 2024, the Nobel Prize in Physiology or Medicine was awarded to Victor Ambros and Gary Ruvkun, who found and characterized microRNAs for the first time in the roundworm *Caenorhabditis elegans* [35,36,37]. Since then, it has been estimated that miRNAs regulate almost two-thirds of all human genes [38]. They are involved in the regulation of various biological processes [39], including cell growth, differentiation, development, and apoptosis [40], and their dysregulation has been associated with human diseases [41] such as diabetes, cardiovascular disorders, and cancer [41,42,43,44,45]. They are potentially useful biomarkers since they can be detected both in tumor tissue and in peripheral blood, and they are stable during sample handling and storage [46]. This review outlines the biogenesis of miRNAs and examines the involvement of miR-155 in the development of DLBCL. It also explores its potential as a diagnostic, prognostic, and predictive biomarker in DLBCL patients.

## 2. Materials and Methods

This is a narrative review. Bibliographic surveys were performed using the main databases (PubMed and Google Scholar). The keywords used for the search linked to all types of articles and included DLBCL, lymphoma correlated to miRNAs, non-coding RNAs, and miR-155. Only articles in English were included. The references of included articles were searched to identify additional studies. There was no time limitation or predefined inclusion or exclusion criteria. A limitation of this study was the non-systematic search of the literature.

## 3. miRNAs Biogenesis

**Figure 1:**
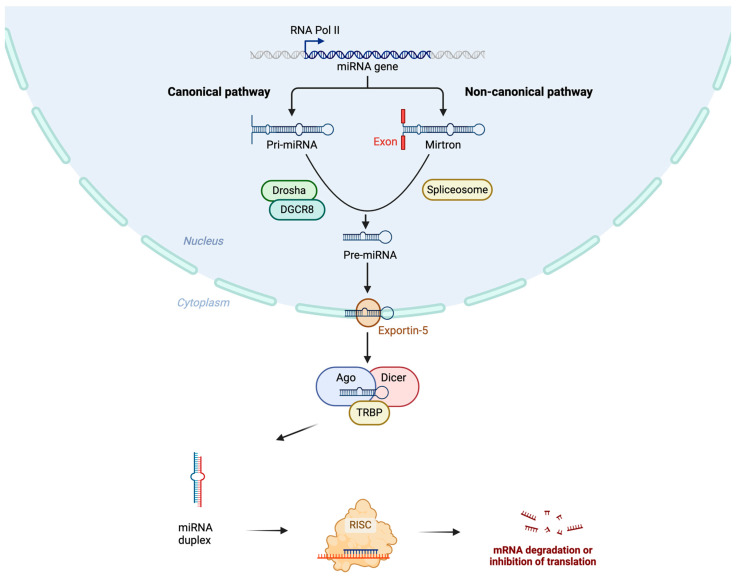
The synthesis of miRNAs. miRNAs are transcribed by polymerase II into primary miRNAs (pri-miRNAs), which are processed in the nucleus by Drosha and its cofactor DGCR8 into pre-miRNAs. These are exported to the cytoplasm by exportin 5, where they are bound by the Dicer/TRBP complex, forming small double-stranded RNAs. The miRNA duplex is then loaded into Argonaute, forming the RISC. The RISC identifies specific mRNA targets, leading to mRNA degradation, destabilization, or translational suppression. Alternative miRNA biogenesis pathways, including Drosha-independent (where the pri-miRNA is spliced by a spliceosome) and Dicer-independent mechanisms, also exist. Ago2: Argonaute 2. DGCR8: DiGeorge syndrome critical region 8. RISC: RNA-induced silencing complex. TRBP: Transactivation response element RNA-binding protein. Created in BioRender. Koumpis, E. (2024) BioRender.com/l31v229 (accessed on 19 October 2024).

miRNAs are preferentially transcribed by polymerase II into primary miRNAs (pri-miRNA) that are subsequently processed in the nucleus by the enzyme Drosha and its cofactor binding protein, DiGeorge syndrome critical region 8 (DGCR8), to generate pre-miRNAs (Figure 1) [47,48,49]. Pre-miRNAs are exported by exportin 5 to the cytoplasm, where they are bound to the RNase Dicer, leading to the formation of small double-stranded RNAs [50]. The miRNA duplex is then loaded into an Argonaute protein, which promotes the assembly of a ribonucleoprotein complex known as the RNA-induced silencing complex (RISC). The RISC is composed of the transactivation-responsive RNA-binding protein (TRBP) and Argonaute 2 (Ago2) [51]. The RISC facilitates the identification of the specific mRNA target by recognizing complementary sequences in the 3′ untranslated region. It can result in mRNA degradation, destabilization, or translational suppression without the need for precise pairing in humans. [40]. Alternative or noncanonical pathways for miRNA biogenesis have been identified, including Drosha-independent and Dicer-independent pathways, where pri-miRNAs are processed to pre-miRNAs by the spliceosome machinery and debranching enzyme [40,52]. The biosynthesis of miRNAs is illustrated in Figure 1.

Since imperfect cleavage of pri- or pre-miRNA can result in diverse 5′ or 3′ ends, a single miRNA gene can produce numerous miRNA isoforms (isomiRs). Different genes and biological pathways can be targeted by distinct isomiRs of the same miRNA. Furthermore, several isomiRs are unique to a particular type of cancer, making them excellent candidates for future biomarkers. [40,53]. MiR-155, and especially miR-155-3p, are the most specific miRNAs for DLBCL [53]. Dysregulation of miR-155 is linked to B-cell lymphomagenesis [54], including DLBCL [46].

## 4. miR-155 and Its Role in DLBCL Pathogenesis

MiR-155 is a conserved multifunctional miRNA transcribed from a non-coding RNA B-cell Integration Cluster (BIC) located on chromosome 21 [55]. It is activated by insertion of the avian leukosis virus (ALV) in the promoter region of the BIC locus in B-cell lymphomas. MiR-155 has been implicated in the regulation of immune responses, hematopoiesis, and inflammation [46,56]. It was shown that miR-155 regulates differentiation of T helper cells and the GC reaction to produce an efficient T cell-dependent antibody response by, at least in part, regulating the production of various cytokines [57]. MiR-155 controls myeloid development and inflammatory cytokine production in myeloid cells through targeting SOCS1 and the Src homology-2 domain containing inositol-5 phosphatase (SHIP1), a negative inhibitor of the PI3K/Akt pathway [58]. Aberrant expression of miR-155 has been associated with both hematologic and solid malignancies [46,59,60]; therefore, it is considered an oncomiR.

The oncogenic nature of this miRNA was confirmed in Eμ-miR-155 transgenic mice, where the mice develop aggressive pre B-cell neoplasms within 3–4 weeks [54]. In addition, in the same animal model, it has been shown that downregulation of Src homology 2-domain-containing inositol 5-phosphatase 1 (SHIP1) and CCAAT Enhancer Binding Protein Beta (CEBPβ) proteins by miR-155 contributed to the development of B-cell neoplasias [61]. SHIP1 and CEBPβ are two important regulators of the IL-6 signaling pathway. Upregulation of miR-155 results in the downregulation of these genes, hence inhibiting B-cell differentiation and causing an improved cell survival due to activation of phosphatidylinositol 3-kinase/protein kinase B (PI3K/Akt) and mitogen-activated protein kinases (MAPK) pathways [61,62,63]. PI3K inhibitors have been examined in clinical trials of patients with relapsed or refractory DLBCL, and these agents could be more effective in patients with miR-155 overexpression [64]. Besides these targets, miR-155 also targets the Human Germinal-center Associated Lymphoma (HGAL) gene. *HGAL* is a GC specific gene that inhibits lymphocyte and lymphoma cell motility by activating the RhoA signaling cascade and interacting with actin and myosin proteins [65,66]. Its expression in patients with DLBCL has been associated with longer OS independently of the IPI score [65]. Overexpression of miR-155 downregulates *HGAL* and may lead to lymphoma cell dissemination and aggressiveness [66].

Splenocytes of Eμ-miR-155 transgenic mice, which overexpress miR-155 in B-cells, express lower levels of IKKβ mRNA than their wild-type counterparts, suggesting that miR-155 may control the expression of both IkappaB kinase beta (IKKbeta; IKKβ) and IkappaB kinase epsilon (IKKepsilon, IKKε), which leads to repression or at least reduced NF-κB activation through a negative feedback loop, repressing or limiting inflammation and immunity [62,67]. MiR-155 most probably directly targets transcripts that code for several proteins involved in LPS signaling, such as the Fas-associated death domain protein (FADD), IKKε, and the receptor (TNFR superfamily)-interacting serine-threonine kinase 1 (Ripk1) while enhancing TNF-alpha (TNF-α) translation, leading to anti-apoptotic effects [68]. In addition, miR-155 targets histone deacetylase 4 (*HDAC*4) and impairs the transcriptional activity of B-cell lymphoma 6 (Bcl-6) [69]. The reduction in Bcl-6 subsequently leads to de-repression of some of the known Bcl-6 targets, like inhibitor of differentiation (Id2), IL-6, c-Myc, Cyclin D1, and Mip1α/ccl3, all of which promote cell survival and proliferation [69]. These findings may have a significant clinical impact on the treatment of miR-155-induced DLBCL, especially with HDAC or Bcl-6 inhibitors, which are not yet used in clinical practice [69,70]. MiR-155 directly targets the gene encoding the bone morphogenetic protein (BMP)-responsive transcriptional factor *SMAD5*, a modulator of transforming growth factor-beta (TGF-β) signaling [71]. In DLBCL models, expression of miR-155 inhibited the activation of the retinoblastoma protein (RB), reducing the abundance of the inhibitory pRB-E2F1 complex and limiting G_0_/G_1_ arrest through the release of E2F1 [72].

**Figure 2:**
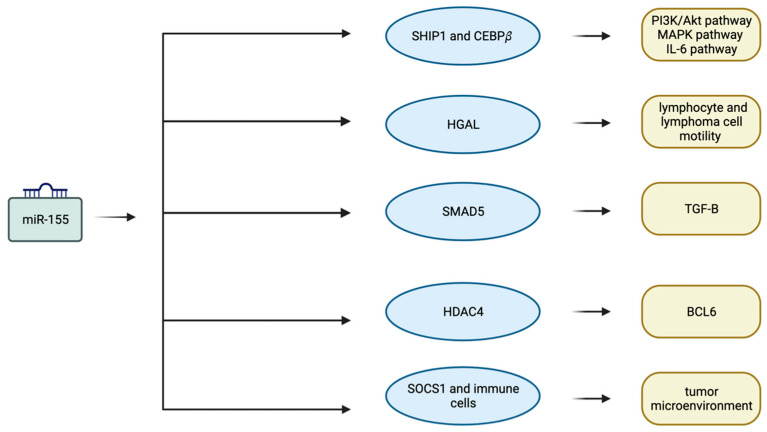
Targets of miR-155 play a crucial role in DLBCL pathogenesis. miR-155 regulates key pathways, transcription factors, and cytokine expression, and it shapes the tumor microenvironment in DLBCL. BCL6: B-cell lymphoma 6. CEBPβ: CCAAT Enhancer Binding Protein Beta. HDAC4: Histone deacetylase 4. HGAL: Human Germinal-center Associated Lymphoma. IL-6: Interleukin 6. MAPK: Mitogen-activated protein kinases. PI3K/Akt: Phosphatidylinositol 3-kinase/protein kinase B. SHIP1: Src homology 2-domain-containing inositol 5′-phosphatase 1. SOCS1: Suppressor of cytokine signaling-1. TGF-β: Transforming growth factor-beta. Created in BioRender. Koumpis, E. (2023) BioRender.com/v57f612 (accessed on 19 October 2024).

MiR-155 also plays a role as a modulator of immune responses [73]. Small lipid extracellular vesicles (EVs), known as exosomes, are thought to have an important role in extracellular communication. MiRNAs, including miR-155, can be involved in exosomes and transferred by them [74,75]. Exosomes with miR-155 are released by the dendritic cells (DCs) and are capable of regulating inflammatory response [75]. The anti-tumorigenic role of miR-155 in DC functionality has been corroborated by both in vitro [76] and in vivo [77] studies, demonstrating that miR-155-enriched exosomes enhance DC-mediated cytokine production and increase tumor infiltration with cytotoxic and helper lymphocytes while reducing regulatory lymphocytes [76,77]. The metastasis of colorectal carcinoma has been linked to the miR-155 exosomal interaction between cancer cells and cancer-associated fibroblasts (CAFs) [78]. Therefore, miR-155 is a crucial determinant in either tumor progression or tumor microenvironment formation in cancer [73]. Furthermore, following their response to danger signals (pathogen-associated molecular pattern (PAMP) or damage-associated molecular pattern (DAMP) molecules such as IFN-β), macrophages and DCs have higher levels of miR-155. This also has an impact on the functions of myeloid-derived suppressor cells (MDSCs), a subset of immature myeloid cells with strong immunosuppressive activity [73,79]. It is noteworthy that the presence of MDSCs in the tumor microenvironment is often associated with poor prognosis [80]. The Foxp3-dependent regulation of miR-155 can confer competitive fitness to regulatory T cell subsets by targeting the suppressor of cytokine signaling-1 (SOCS1) protein [81]. Moreover, miR-155 modulates the generation of immunoglobulin class-switched plasma cells via targeting PU 1 and activation-induced cytidine deaminase (AID) [82,83]. Figure 2 displays key targets of miR-155 that are significant in the development of DLBCL.

Given the involvement of miR-155 in DLBCL pathogenesis through its regulation of multiple pathways, it holds potential as a biomarker for clinical assessment in the future.

## 5. miR-155 as a Biomarker in DLBCL

A biomarker is an objectively measured and evaluated indicator of normal biological or pathogenic processes or pharmacologic responses to a therapeutic intervention [84]. A diagnostic biomarker detects or confirms the presence of a disease or condition of interest or identifies an individual with a subtype of the disease [85]. The predictive value of biomarkers assesses the impact of therapy on a patient with a particular condition, whereas their prognostic value predicts the outcome regardless of the treatment approach [84,86]. In other words, a prognostic biomarker provides information about a likely cancer outcome independent of any treatment received, while a biomarker is predictive if the treatment effect (experimental compared with control) is different for biomarker-positive patients compared with biomarker-negative patients [87].

**Figure 3:**
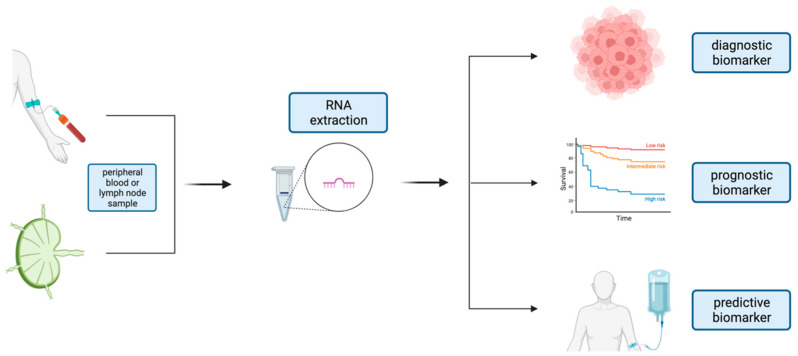
miRNAs as a non-invasive tool in patients with DLBCL. miRNAs can be extracted directly from lymph node tissue or obtained easily from peripheral blood (liquid biopsy), rendering them valuable biomarkers that can serve as potential diagnostic, prognostic, or predictive biomarkers. Created in BioRender. Koumpis, E. (2023) BioRender.com/m16t064 (accessed on 19 October 2024).

To improve the prognosis assessment and treatment response evaluation in patients with DLBCL, various research groups have focused on identifying and exploring novel biomarkers. Invasive techniques for cancer diagnosis and monitoring are gradually being replaced by non-invasive approaches like liquid biopsies [88]. While circulating tumor cells can offer tumor-specific information, their analysis is less appealing in DLBCL because this lymphoma type typically lacks circulating tumor cells [89]. The clinical application of cell free RNAs such as miRNAs and circRNAs has shown promise as a precision medicine biomarker in DLBCL [32]. MiR-155 is a potential diagnostic, prognostic, and predictive biomarker of DLBCL [46]. As a candidate biomarker, the expression of miR-155 has been studied both in tissues and in peripheral blood (Figure 3).

### 5.1. miR-155 as a Diagnostic Biomarker

Studies evaluating miR-155 as a diagnostic biomarker in tissue samples and peripheral blood have yielded conflicting results [90]. Many studies have observed an increase in miR-155 expression in the neoplastic tissue or serum of patients with DLBCL compared to non-neoplastic lymph nodes and peripheral B-cells [91,92,93,94,95,96,97,98,99], as well as the serum/plasma or serum-derived exosomal vesicles [100,101,102,103,104,105] of healthy controls. On the other hand, several studies did not confirm any differential expression of miR-155 between DLBCL patients and controls [99,106,107,108,109]. Serum levels of miR-155 have shown a positive correlation with its expression in tissue samples, probably underlying the usefulness of this molecule as an easily accessible biomarker in peripheral blood [102,105]. Interestingly, the serum levels of miR-155 might show an expression pattern that is dependent on the natural course of the disease, with fluctuation at diagnosis, while on treatment or during progression [110]. A comparison of all miRNA levels between patients during progression and those undergoing treatment reveals a decrease in miRNA levels [110].

Overexpression of miR-155 along with downregulation of PU1 was detected not only in DLBCL but also in tissues from other lymphoid malignancies [91]. In a study that included DLBCL (58 cases) and FL (46 cases) tissue samples, miR-155 was overexpressed in both of them, discriminating the lymph nodes affected by the tumor from the non-neoplastic ones [94]. In another large study, including 200 tissue samples of DLBCL, there was strong evidence of the diagnostic role of miR-155 overexpression in DLBCL [92]. It was found that miR-155 was overexpressed in de novo DLBCL (*n* = 35), transformed DLBCL (*n* = 14), and follicular center lymphoma cases (*n* = 27) compared to normal B-cells [95].

Data have been more convergent as to the diagnostic value of miR-155 in the distinction between ABC and GCB subtypes of DLBCL. Numerous original studies have shown that tissue or serum miR-155 expression levels are upregulated in patients with a non-GCB/ABC subtype compared with GCB [92,93,95,96,98,103,111,112,113], probably because of the targeting of *HGAL* by miR-155, as previously discussed [66]. MiR-155 levels also seem to be significantly different between de novo DLBCL and transformed aggressive B-cell lymphoma [111]. Specifically, miR-155 was found at higher levels in de novo DLBCL cases in comparison with transformed DLBCL cases [111]. Nevertheless, in a separate study, it was demonstrated that the transformation of FL to DLBCL was linked to changes in miRNA expression (including miR-223, 217, 222, 221 and let-7i and 7b). [98]. However, miR-155 was not included in the list of miRNAs that were differently expressed between de novo and transformed DLBCL [98]. Consequently, miR-155 levels cannot be used to access the differentiation of FL from DLBCL or the prediction of the transformation to DLBCL [98].

Prospective randomized studies are necessary to further examine and delineate a potential diagnostic role of miR-155 in patients with DLBCL as well as standardized protocols for miRNA measurement and analysis.

### 5.2. Prognostic Value of miR-155

There is a debate over whether miR-155 has the potential to be a prognostic biomarker in DLBCL patients. In a study that reviewed 90 DLBCL tissue samples, it was demonstrated that a longer 5-year PFS was associated with reduced levels of miR-155 expression in de novo DLBCL cases [73]. In addition, two other studies showed that the upregulation of miR-155 was associated with poorer survival [113,114]. Detection of the expression levels of miR-155 in tumor tissues of 118 lymphoma patients by real-time polymerase chain reaction (PCR) resulted in the dichotomy of patients based on the median level of miR-155 expression in patients with high and low expression of miR-155 [114]. It was found that patients with high expression of miR-155 had a shorter survival than the group with low miR-155 expression [114]. However, in another study, high levels of miR-155 were linked to better clinical outcomes in patients with DLBCL of the GCB subtype who received R-CHOP treatment, regardless of their IPI score [115].

The prognostic value of miR-155 has also been studied as a measurable biomarker in peripheral blood, and it was concluded that serum miR-155 levels do not have prognostic significance [100]. However, other studies that were published later indicated that higher levels of miR-155 in peripheral blood were associated with worse prognosis independently of the IPI score [103,104,105].

As it was also referred in the diagnostic value of miR-155, prospective randomized studies are necessary to delineate the potential prognostic role of miR-155 in patients with DLBCL in order to reduce the discrepancies. For the same reason, a consensus on standardized protocols for miRNA measurement and analysis is considered essential.

### 5.3. miR-155 as a Predictive Biomarker

The predictive value of a biomarker provides information about the effect of therapy on a patient with a certain condition. MiR-155 seems to be a promising predictive biomarker that can be detected in both tissue and peripheral blood. For example, the expression of miR-155 was found to be an independent predictor for selecting chemotherapy protocols in de novo DLBCL, serving as a tissue biomarker with predictive value [93]. Patients with high miR-155 expression who received R-CHOP had improved OS and PFS compared to those on the CHOP treatment [93]. It is noteworthy that this difference was not observed with low expression of miR-155 [93]. In another study, high expression of miR-155 was associated with R-CHOP treatment failure [113]. More recently, it was found that increased miR-155 expression enhanced vincristine sensitivity in DLBCL cell lines by targeting the *WEE1* gene [115].

A study was conducted to evaluate the relationship between circulating miRNA expression, chemoresistance, and prognosis in DLBCL patients [102]. It was first shown that there was a significant correlation between miRNA levels in serum and formalin-fixed, paraffin-embedded (FFPE) tissues, suggesting that circulating miRNAs could serve as effective, non-invasive biomarkers reflecting miRNA levels in tumor tissue [102]. Next, the expression of eight miRNAs was analyzed in 56 DLBCL patients, and the continuous monitoring of circulating miR-125b and miR-130a levels showed their involvement in the recurrence, progression, and chemoresistance of DLBCL patients [102]. However, peripheral blood levels of miR-155 did not correlate with chemosensitivity or chemoresistance [102]. Nevertheless, in a recent study, it was shown that exosomal miR-155 was overexpressed in refractory/relapsed patients compared to responsive patients and patients receiving R-CHOP [116]. On the other hand, it was found that miR-155 levels in serum-derived extracellular vesicles were significantly different in certain hematologic malignancies compared to controls, but not in DLBCL [109].

## 6. miR-155 in Solid Tumors and in Hemato-Oncology

Apart from DLBCL, the value of miRNA-155 as a diagnostic, prognostic, or predictive biomarker, or even as a potential therapeutic target, has been investigated in several malignancies, with rather contradictory results being found in most cases.

A recent meta-analysis addressing the prognostic role of miR-155 in patients with breast cancer concluded that the data remain controversial, although up to 90% of original studies correlated with increased serum or tumor tissue miR-155 expression with worse disease characteristics and outcomes, supporting the oncogenic role of this miRNA [117]. Interestingly, it was shown that elevated tissue expression of miR-155 by breast tumor cells increases anti-tumor T cell invasion, sensitizes malignant cells to immune checkpoint blockade therapy and correlates with better patient outcomes [118]. Similarly, induced overexpression of miR-155 followed by olaparib exposure was found to further reduce tumor cell survival compared to olaparib alone, indicating the potential of miR-155 to predict response to PARP1 inhibition therapy in breast cancer patients [119]. In contrast, miR-155 was shown to induce paclitaxel resistance in breast cancer cells by increasing their viability, while its inhibition re-sensitized paclitaxel-resistant tumor cells, thus pointing to a novel targeting therapeutic approach [120].

In colorectal cancer (CRC), data about the utility of miR-155 as a biomarker have generally been conflicting. Some studies have found that miR-155 is upregulated in the tumor tissue of patients with CRC and it associated with worse survival rates, supporting its role as a diagnostic and prognostic biomarker [121,122]. Studies supporting the oncogenic potential of miR-155 have demonstrated that it can promote cell proliferation and metastasis development by activating cancer-associated fibroblasts or direct binding to HuR, a nuclear RNA binding protein [78,123,124]. Contrary to these results, other studies have shown that miR-155 is reduced in CRC tissues and cell lines [125]. It was observed that miR-155 can impede the advancement and spread of CRC by suppressing CTHRC1 (collagen triple helix repeat containing 1), a protein associated with cancer growth [125]. In relation to therapy, overexpression of miR-155 seems to increase the chemoresistance of tumor cells to cisplatin, potentially by downregulating forkhead box O3 (FOXO3), a tumor suppressor transcription factor which could possibly be exploited for the treatment of drug-resistant CRC [126]. Finally, although several miRNA panels were found useful by a systematic review and meta-analysis for CRC screening, miR-155 was not included in any of these panels [127].

Lung cancer studies on miR-155 provide contradictory results about its use as a biomarker. Several studies have determined that the expression levels of miR-155 are increased in individuals with non-small-cell lung cancer (NSCLC) and are associated with the stage of the disease. MiR-155 was also identified as a negative regulator of FOXO3a in NSCLC, leading to increased gefitinib resistance and lung cancer stemness in vitro and in vivo [128]. There is evidence suggesting that this miRNA targets genes that are involved in cell proliferation or tumor suppression, such as *PTEN* and *FGF9*. Additionally, inhibiting miR-155 can lead to autophagic cell death of tumor cells, indicating a potential avenue for therapeutic intervention. [129,130,131]. Additional studies did not find a significant difference in miR-155 expression between NSCLC patients and controls, while a systematic review concluded that miR-155 is effective as a diagnostic but not as a prognostic biomarker [132,133].

Finally, in head and neck carcinomas, a recent systematic review with meta-analysis confirmed the negative effect of high miR-155 expression in patients’ survival [134].

Among hematologic malignancies, the prognostic role of miR-155 has been studied in both myeloid and lymphoid malignancies. A systematic review with meta-analysis of 18 studies revealed that miR-155 has a significant role in predicting patient outcomes in hematologic malignancies [135]. The study found that increased expression of miR-155 was consistently linked to lower survival rates (OS, PFS, event-free survival) in patients with various types of hematologic malignancies, such as acute leukemias, chronic lymphocytic leukemia (CLL), and lymphomas [135].

Additionally, in acute myeloid leukemia (AML), higher expression levels of miR-155 have been associated with *FLT3-ITD* (FMS-like tyrosine kinase-3 internal tandem duplication) gene mutation, and several ongoing studies have investigated the role of miR-155 in minimal residual disease (MRD) monitoring or as a future therapeutic target [136]. In patients with myelodysplastic neoplasms, aberrant expression of miR-155 may promote the progression to AML, and its levels seem to be prognostic of shorter survival [137,138]. Even in chronic myelogenous leukemia (CML), miR-155 was found to be one of the most commonly upregulated miRNAs in a most recent systematic review [139].

In terms of lymphoid malignancies, CLL and DLBCL have gathered the most research interest, given their higher incidence. MiR-155 was found to be significantly higher in patients with monoclonal B-cell lymphocytosis (MBL) compared with controls but lower compared with CLL patients, which might be useful for monitoring MBL patients. Higher levels of miR-155 among CLL patients were associated with more aggressive disease characteristics and a worse prognosis. Several findings also highlighted the potential predictive role of miR-155, as its levels were found to be lower in pre-treatment samples of patients who showed higher response rates to different therapies [140,141,142]. Therapeutic targeting of miR-155 with the anti-miR Cobomarsen, in patients with CLL is under investigation following encouraging results in a small number of patients with cutaneous T cell lymphoma [143]. Furthermore, Cobomarsen could be a potential future therapeutic tool in DLBCL [144]. Cobomarsen reduced cell proliferation and induced apoptosis in ABC-DLBCL cell lines [144]. When administered intravenously in a xenograft immune-compromised NSG (NOD Scid Gamma) mouse model of ABC-DLBCL, Cobomarsen decreased the tumor volume, initiated apoptosis, and de-repressed direct miR-155 target genes [144]. In this study, a patient who underwent five cycles of Cobomarsen treatment experienced effective reduction and stabilization of the tumor growth with non-toxic effects [144].

## 7. Conclusions and Future Research

MiR-155 is an oncomiR that significantly contributes to cancer development through many pathways [145]. Moreover, the fact that miR-155 is the most specific microRNA that is dysregulated in DLBCL (especially the isomiR miR-155-3p) renders this microRNA a potential diagnostic, prognostic, and predictive biomarker [53]. ΜiR-155 level measurement is a non-invasive tool that can be incorporated in everyday clinical practice, especially to determine clinical outcomes or specific treatment responses in the future. Future research should evaluate miR-155 levels in the recently described DLBCL clusters to assess their diagnostic importance. With the emergence of new therapy regimens like Pola-RCHP [22], the predictive usefulness of miR-155 should be reassessed. Several retrospective studies with limited sample sizes, and a lack of a standardized protocol for miRNA measurement, reported conflicting findings. More extensive investigations and a consensus on standardized protocols for miRNA measurement and analysis are needed to determine the possible diagnostic, prognostic, and predictive significance of miR-155 in relation to the updated classifications of DLBCL and emerging treatment strategies.
